# Improving the Surface Integrity of 316L Steel in the Context of Bioimplant Applications

**DOI:** 10.3390/ma16093460

**Published:** 2023-04-28

**Authors:** Krzysztof Szwajka, Joanna Zielińska-Szwajka, Tomasz Trzepieciński

**Affiliations:** 1 Faculty of Mechanics and Technology, Department of Integrated Design and Tribology Systems, Rzeszow University of Technology, ul. Kwiatkowskiego 4, 37-450 Stalowa Wola, Poland; 2 Faculty of Mechanics and Technology, Department of Component Manufacturing and Production Organization, Rzeszow University of Technology, ul. Kwiatkowskiego 4, 37-450 Stalowa Wola, Poland; j.zielinska@prz.edu.pl; 3 Department of Manufacturing Processes and Production Engineering, Rzeszow University of Technology, al. Powstańców Warszawy 8, 35-959 Rzeszów, Poland; tomtrz@prz.edu.pl

**Keywords:** AISI 316L, bioimplant, hardness, turning, surface integrity, surface topography

## Abstract

Bioimplants should meet important surface integrity criteria, with the main goal of the manufacturing process to improve wear and corrosion resistance properties. This requires a special approach at the cutting stage. During this research, the impact of the cutting parameters on improving the surface integrity of AISI 316L steel was evaluated. In this context of bioimplant applications, the mean roughness Sa value was obtained in the range of 0.73–4.19 μm. On the basis of the results obtained, a significant effect was observed of both the cutting speed and the feed rate on changes in the microstructure of the near-surface layer. At a cutting speed of 150 m/min, the average grain size was approximately 31 μm. By increasing the cutting speed to 200 m/min, the average grain size increased to approximately 52 μm. The basic austenitic microstructure of AISI 316L steel with typical precipitation of carbides on the grain boundaries was refined at the near-surface layer after the machining process. Changing the cutting speed determined the hardness of the treated and near-surface layers. The maximum value of hardness is reached at a depth of 20 μm and decreases with the depth of measurement. It was also noted that at a depth of up to 240 μm, the maximum hardness of 270–305 HV1 was reached, hence the height of the machining impact zone can be determined, which is approximately 240 μm for almost all machining conditions.

## 1. Introduction

The processing of biocompatible materials requires improvement due to the significant impact of the quality of the treated surface on compatibility with human organs. The specific requirements in terms of bioimplants are analyzed and described in the scientific literature. A high strength-to-weight ratio, good mechanical properties and high corrosion resistance are the general requirements a material must have to be considered biocompatible [1]. Moreover, the properties desired in bioimplant application include biocompatibility with human tissues [2]. Stainless steel and titanium implants are more desirable for different anatomic locations [3]. Stainless steel is significantly stiffer than bone [4]. The use of titanium alloys is associated with greater susceptibility to fretting corrosion and a higher price compared to implant steel [5]. 316L stainless steel, due to its widespread availability, low cost and ease of production, is among the most commonly used biomaterials [6]. 316L steel has been employed in the design of implants for orthopedic care: rigid intramedullary nails, sliding hip screws, screws, orthopedics plates and flexible nails.

Titanium and its alloys are categorized as bio-inert, while 316L stainless steel is categorized as bio-tolerant [7]. The implants manufactured from 316L steel are cheaper than titanium alloys [8]. Nickel stabilizes the formation of austenite in the stainless steels. [9]. Barber et al. [3] pointed out that there is no clear answer to which of these materials is generally better. However, both of these materials have properties that may make them better in certain anatomical locations.

Machining processes, more specifically turning and milling, are common for finishing the usable surface. Turning is a mechanical process for treating a surface. It leads to deep modifications of the processed material layer (residual stresses and microstructure)—up to several hundred micrometers in certain cases [10,11]. According to Rech et al. [12], the term surface integrity aims to describe the state of a surface with regard to its potential performance. Over the past ten years, much attention has been devoted to predicting turning-induced surface integrity using combined experimental and computational approaches for different materials [13]. Both the topography and the residual stresses have been shown to have an effect on the number of cycles to failure [14]. Machining processes are associated with the occurrence of friction and a significant increase in temperature in the processed layer of the workpiece, which can, in some cases, lead to the development of cracks, phase transitions or certain microstructural defects near the surface [15]. The tensile stress field increases as a result of heat generated during cutting [16]. Therefore, microstructural changes and thermal gradients usually resulting from turning operations are a major concern regarding the service life of products [17].

Titanium, magnesium and its alloys are considered the primary metals for dental joints [18], cardiovascular [19] and orthopedic [20] implants. AISI 316L is primary stainless steel, where “L” means low carbon content and 316 is the metal grade suitable for implantation, often referred to as surgical stainless steel [21]. 316L steel is a widely used implant material for internal fixation because it can be bent and formed into the implant [22]. Although the composition of this alloy may vary slightly, AISI 316L stainless steel is characterized by a chromium and nickel content of 18% and 8%, respectively. In addition, nickel (12%) is added to obtain an austenite microstructure. This microstructure is characterized by better plasticity compared to the basic crystalline structure of ferrite. AISI 316L is easy to machine because there is less violent martensite formation during the cutting operation [23], making it easier to machine with conventional machine tools. The general requirement of the mean roughness Ra for implants ranges from 1.5 to 4 μm, depending on bioimplant application [24]. Rønold et al. [25] suggested that the optimal arithmetic mean deviation for bioimplants should be in the range between 3.62 and 3.90 μm. In addition, lower values of material hardness and reduced wear resistance may lead to inflammation in the body [26].

In the literature, studies can be found concerning various analyses related to the surface integrity of the machined surface of AISI 316L steel. Kadi et al. [27] experimentally analyzed a dry turning process to minimize surface roughness and maximize microhardness for AISI 316L. The optimum mean roughness (Ra = 1.66 μm) and the maximum microhardness (335 HV) were obtained. Ali et al. [28] machined 316L steel using a ZTA-MgO cutting tool. Increasing the cutting speed (CS) resulted in a decrease in surface roughness. An inverse relationship was observed for the effect of feed rate (FR). Tamayo et al. [29] used artificial neural networks and multiple regression to predict the mean roughness of dry turning 316L steel using GC1115 and GC2015 inserts. It was found that decreasing the feed rate resulted in a reduction in the mean roughness Ra. Basmaci [30] analyzed cutting forces and surface roughness when turning 316L steel. It was observed that mean roughness increases in direct proportion to the increasing feed rate. Tekiner and Yeşilyurt [31] concluded that the mean roughness Ra increases with the increasing feed rate. Toggui et al. [32] investigated the influence of the feed rate and the cutting speed on mean roughness (Ra) when dry turning AISI 316L stainless steel using a GC1525 cermet insert. The results revealed that the mean roughness is mostly affected by the feed rate. Similar results were found by Nur et al. [33] and Ay et al. [34]. Dambare et al. [35] conducted wet and dry turning of 316L steel. They used a Taguchi L9 orthogonal array to analyze the influence of turning parameters on the mean roughness. It was found that the mean roughness Ra decreases with the decreasing feed rate and cutting speed.

In addition to the appropriate physico-chemical properties of the bioimplant material, in medical applications, its surface should be characterized by appropriate surface integrity. This article focuses on ensuring appropriate machining parameters to obtain a surface layer with low roughness and high hardness. The purpose of this work was to analyze the effect of selected cutting parameters on surface roughness when machining AISI 316L steel. Three different cutting speeds and feed rates were considered. Due to the context of the application of the research results to the processing of bioimplants, this research was focused on the impact of machining parameters on the surface roughness surface finish, the hardness and the microstructure of the workpieces. The parameters affecting the machining process (cutting speed v_c_ and feed rate f) were organized by the Taguchi experimental design. To develop the test plan, the range of parameters recommended by the manufacturer of the cutting insert (ISCAR) for machining of 316L steel was used. The recommended cutting parameters are as follows: f_min_ = 0.05 mm/rev, f_max_ = 0.3 mm/rev. However, the recommended cutting speeds are in the range between 150 m/min and 200 m/min.

## 2. Methods and Materials

The details of the research plan and the description of the analysis of the research results obtained are presented in the following subsections.

### 2.1. Materials

**Figure 1:**
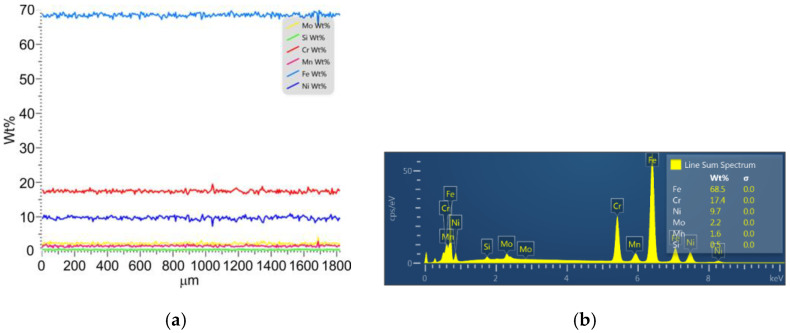
Spectral analysis of AISI 316L steel: (**a**) wt% content of the main elements and (**b**) spectra.

AISI 316L steel was used as the workpiece. It is an austenitic steel containing 2–3% molybdenum. A spectral analysis (Figure 1) of the tested material was carried out using a MIRA3 scanning electron microscope (SEM). The chemical constitution of 316L is given in Table 1. The EDS method is not suitable for accurate carbon measurement, the chemical composition of AISI 316L steel is provided only as informational. The thermos-mechanical properties of this steel that affect its machinability are presented in Table 2. The modulus of elasticity and tensile strength were determined using a uniaxial tensile test. Hardness was measured using the universal Vickers test. The stationary method was used to determine the thermal conductivity of 316L steel.

**Figure 2:**
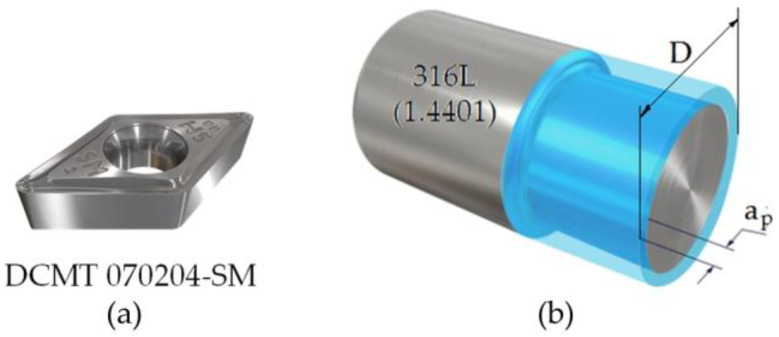
(**a**) Cutting edge and (**b**) workpiece.

In the turning operation, a round bar with a diameter of 22 mm was used as a workpiece, which was turned longitudinally over a length of 40 mm (Figure 2b). The turning operation was carried out at the Gildemeister Twin 32 CNC machining center (Drehmaschinen GmbH, Bielefeld, Germany) using a DCMT 070204-SM turning insert (Figure 2a). Experimental cutting tests were carried out for nine sets of cutting parameters, according to the Taguchi plan, using the L9 orthogonal system with three repetitions. The feed rate and the cutting speed were used in this work, as shown in Table 3. Cutting tests were carried out without the use of a cooling medium (dry machining) and for a depth of cut of 1.5 mm.

### 2.2. Cutting Procedure

The measurement of the surface topography was carried out using a CNC profilometer Hommel-Etamic T8000RC. Measurement of the Ra surface roughness parameter was carried out in accordance with the ISO-4288:1998 standard.

**Figure 3:**
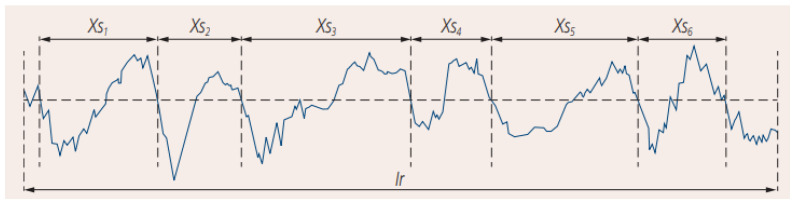
The spacing *X_si_* of the profile elements.

The test guidelines were adopted in accordance with EN ISO 4288. The value of the mean width RSm (Figure 3) in the measurements was 0.13 < RSm ≤ 0.4.

**Figure 4:**
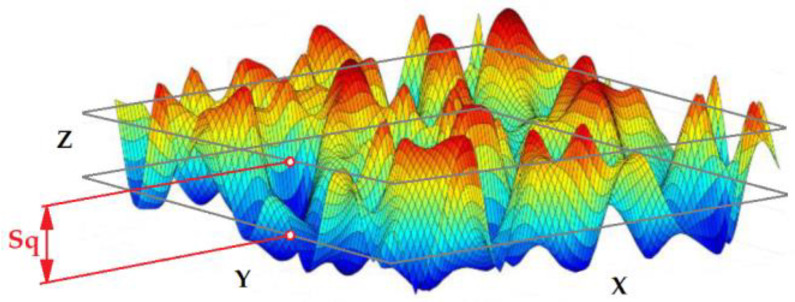
Root mean square height Sq, prepared on the basis of [37].

The S_q_ parameter is defined as the root mean square value of the surface asperities and valleys z(x,y) (Figure 4), within the sampling area. The Sq parameter is included in the ISO 25178-2 [36] standard.

**Figure 5:**
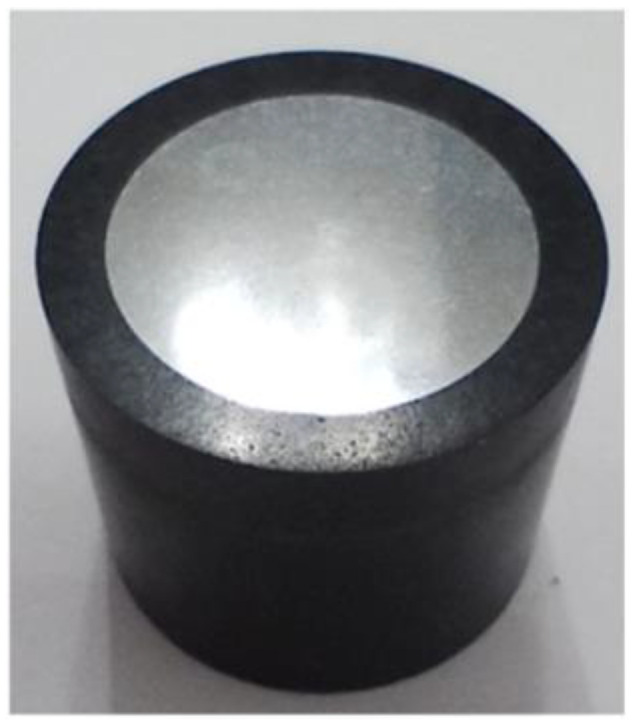
Sample included.

A BP95d electro-erosion cutter wasused to cut specimens. The cut samples were embedded in epoxy resin (Figure 5). Specimens for metallographic examinations were wet polished by 120~2500-grit paper. Finally, diamond paste (grain size 1 μm and 3 μm) was used to polish the surface of specimens. Mi-19 Fe reagent (chemical constitution: 20 g HCl, 90 mL C_2_H_5_OH, 3 g FeCl_3_) was used to etch the specimens. Hardness measurements were also performed on samples prepared in the same way.

The microstructure of the workpiece was analyzed using microscopes on the cross-section of the specimens. Metallographic observations were carried out using an Olympus BX51M (Olympus, Tokyo, Japan) optical microscope coupled with a computer with Olympus Stream Essentials software with magnifications from 50× to 1000×.

**Figure 6:**
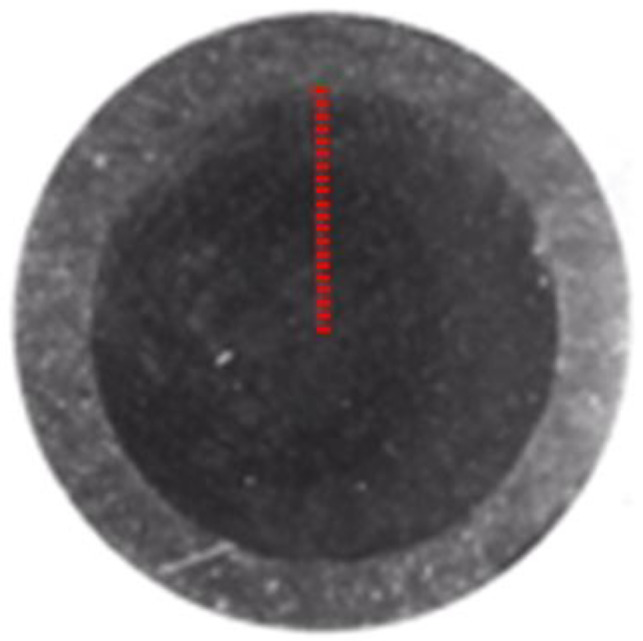
Hardness measurement points on the ANSI 316L.

Measurements of the hardness distribution were made at 20 points along the radius (Figure 6). The distance between points was 50 μm. The first measurement point was located at a depth of 20 μm. The measurements were carried out using the Vickers method on a Qness 60M hardness tester (Qness, Mammelzen, Germany) according to the ISO 6507 standard. A load of 9.807 N means HV 1 (low-force Vickers hardness test) and a test time of 10 s was applied.

## 3. Results

### 3.1. Hardness

**Figure 7:**
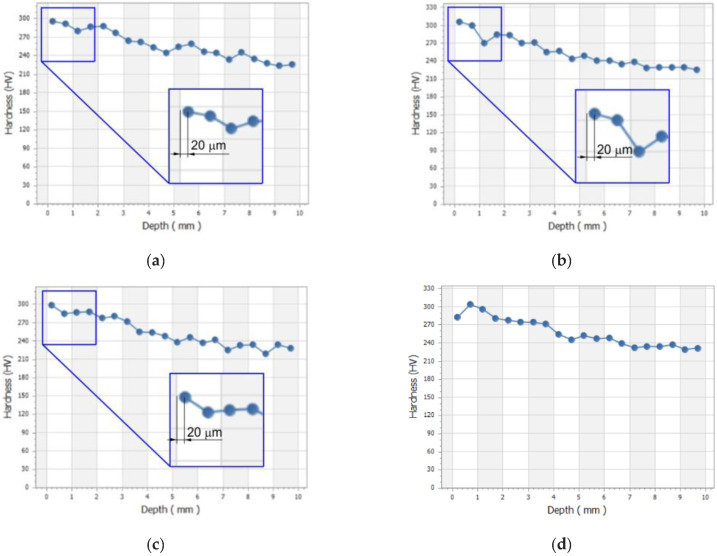

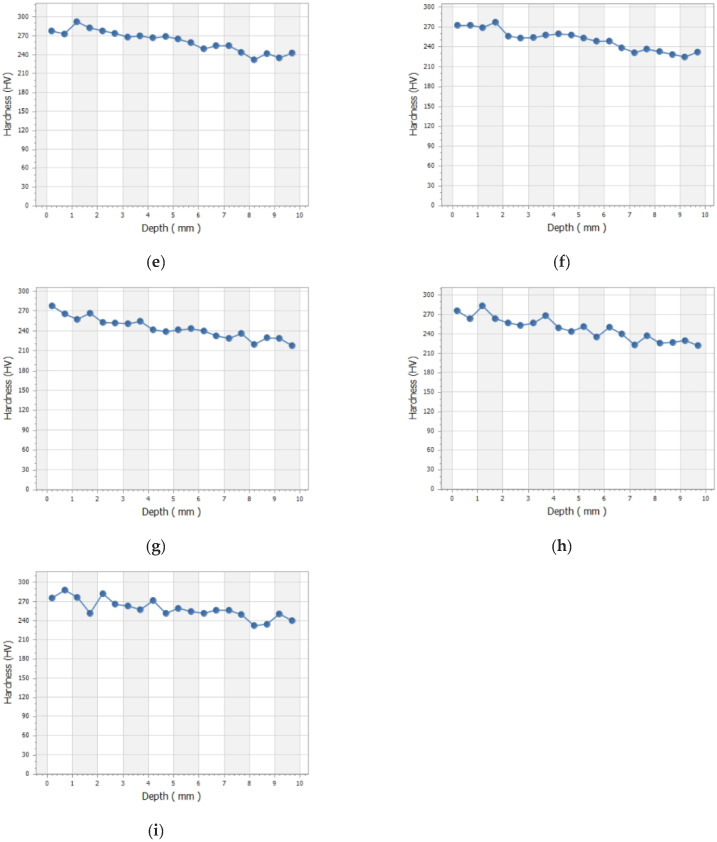
Hardness in the cross-section of the AISI 316L bars machined at the following conditions: (**a**) v_c_ = 200 m/min; f = 0.3 mm/rev, (**b**) v_c_ = 200 m/min; f = 0.15 mm/rev (**c**) v_c_ = 200 m/min; f = 0.08 mm/rev, (**d**) v_c_ = 170 m/min; f = 0.30 mm/rev, (**e**) v_c_ = 170 m/min; f = 0.15 mm/rev, (**f**) v_c_ = 170 m/min; f = 0.08 mm/rev, (**g**) v_c_ = 150 m/min; f = 0.30 mm/rev, (**h**) v_c_ = 150 m/min; f = 0.15 mm/rev, (**i**) v_c_ = 150 m/min; f = 0.08 mm/rev.

Before testing the machinability of AISI 316L steel, its hardness was measured. The mean value of the hardness of the workpiece material was 220 HV1. Figure 7 shows the change in hardness for all the specimens tested. It was found the maximum value of hardness for the CS v_c_ = 200 m/min and the FRs f = 0.08–0.3 mm/rev is reached at a depth of 20 μm and then decreases (Figure 7a–c). At a depth of 240 μm, the limit hardness of 270–305 HV1 (maximum hardness achieved in a given cutting test) was reached. For this hardness, a machining influence zone can be determined, which is approximately 240 μm. The maximum hardness value of AISI 316L is 308 HV1, which is an increase of approx. 40% compared to the value of the base material.

When machining at 200 m/min with three feed rates, it can be seen that the maximum hardness was found at a depth of approximately 20 µm from the machined surface. It should also be noted that the machining was carried out without coolant, which entails the generation of heat in the contact zone. This situation led to near-surface hardening of the machined surface. At CSs of 150 m/min and 170 m/min and a FR value of 0.3 mm/rev, there is a similar trend of increase in hardness. However, at a CS of 170 m/min and a FR value of 0.3 mm/rev, the hardness at a depth of approximately 20 μm was not the maximum value. This may be caused by an increase in the value of the cutting ratio Λ_h_, which has a significant impact on the increase in grain size of the workpiece material.

This was achieved under dry machining conditions at a high CS v_c_ = 200 m/min and a FR f = 0.30 mm/rev. The work hardening is a phenomenon accompanying machining, which was reported by many researchers [38,39]. High cutting speeds and feeds generate more heat, which is responsible for plastic deformation. The influence of FR on work hardening phenomenon was also reported by Khan et al. [40] and Ulutan et al. [41]. Moreover, Khan et al. [40] found the high FR is among the causes of the work hardening of D2 high-carbon tool steel.

**Figure 8:**
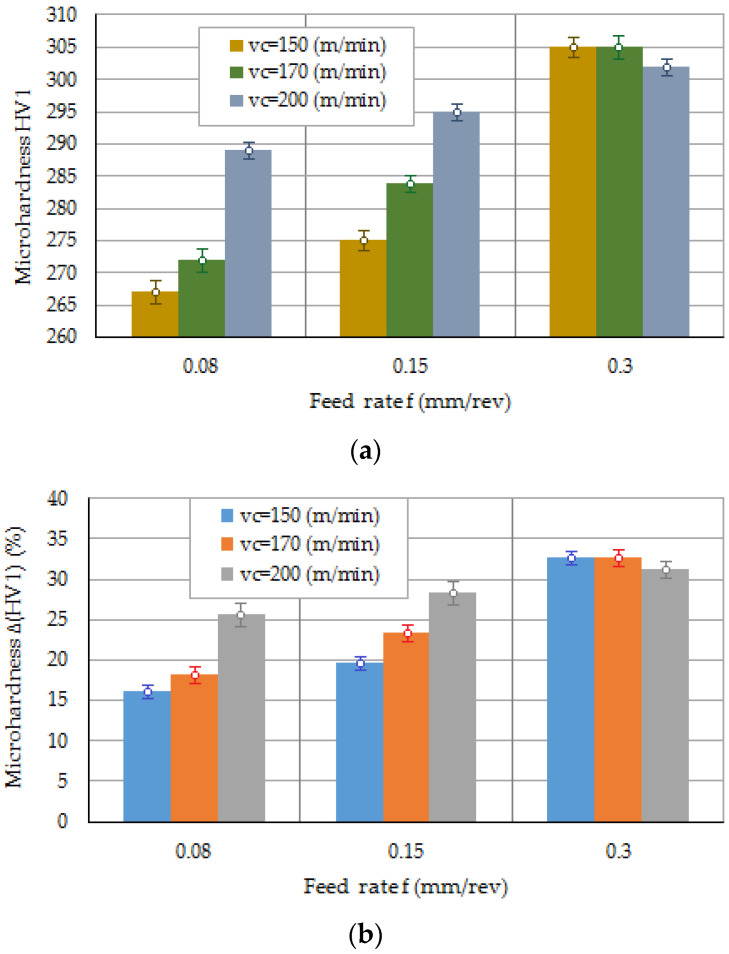
(**a**) Hardness and (**b**) relative increase in the hardness of the surface of AISI 316L steel.

Figure 8 shows the values obtained for the hardness HV1 of the AISI 316L machined surface. It was observed the FR significantly affects the hardness of machined surface (Figure 8a). The influence of the CS on hardness was noticeable only for small feed values. As the FR increased, the effect of the CS on hardness was negligible.

Figure 8b, in turn, shows the percentage increase ΔHV1 in the hardness value that occurred during machining with the cutting parameters adopted in the tests. The parameter ΔHV1 was determined as a ratio of the hardness measured at a depth of 20 μm below the surface HV1_20_ to the hardness of the base metal HV1_BM_:

(1) $$∆HV1=\frac{{HV1}_{20}-{HV1}_{BM}}{{HV1}_{BM}}\cdot 100\%$$

The maximum values of the percentage increase in the hardness of the machined surface were found for the CS v_c_ = 150 and v_c_ = 170 m/min, and the FR f = 0.3 mm/rev. As the CS increases, the chip becomes continuous and makes it difficult to dissipate heat from the cutting zone to the environment.

Machining parameters affect the change in hardness in a thin layer just below the machined surface. Changes in hardness in the area of impact of the cutting edge are caused by changes in microstructure, residual stresses, and differences in heating and cooling rates. The highest percentage increase in hardness in relation to the initial hardness occurred for the highest FR of 0.3 mm/rev. However, it can be seen that the influence of CS for this case is unnoticeable. The hardness of the workpiece machined at FR of 3 mm/rev and various CSs is similar. A different situation occurs for FRs of 0.08 mm/rev and 0.15 mm/rev, then the effect of CS on hardness is important.

### 3.2. Surface Topography Analysis

**Figure 9:**
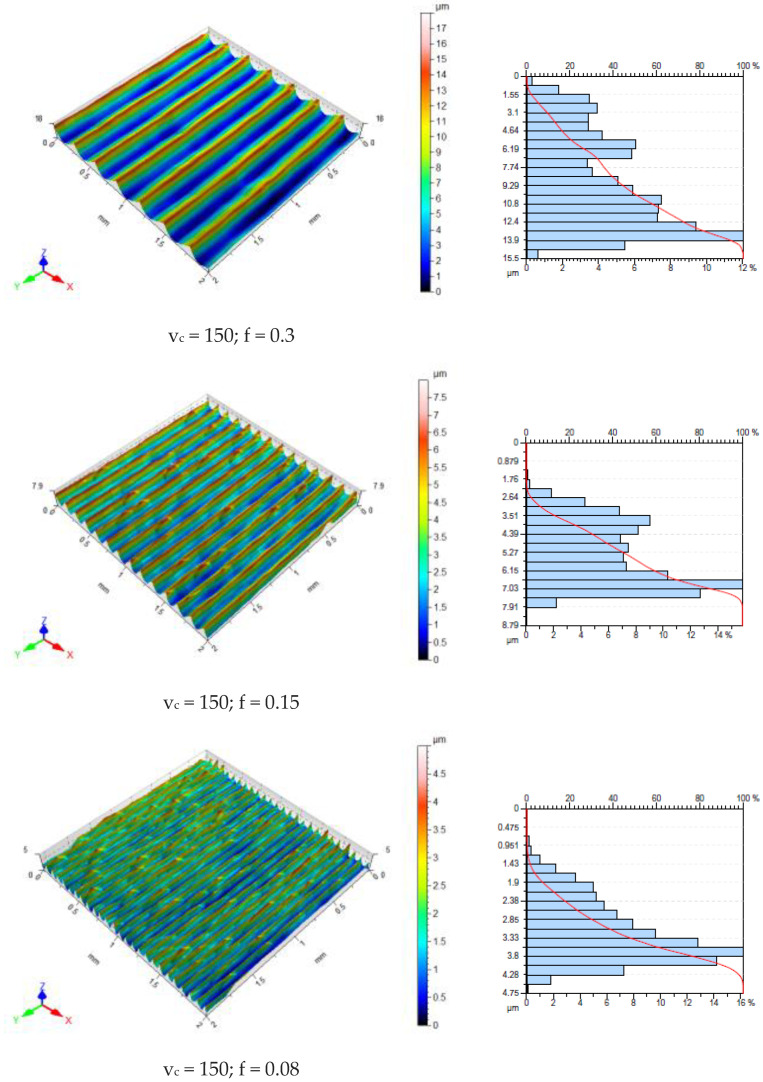
Topography of the machined surfaces (**left**) and bearing area curve (**right**) of AISI 316L steel bars (units: v_c_—m/min, f—mm/rev).

Machined surfaces are evaluated based on surface topography measurements. The surface roughness parameter Ra is susceptible to individual summits and valleys [42]. It is, therefore, only of minor importance. To assess surface roughness in industrial conditions, the mean roughness Ra is most often used. Figure 9 shows the surface topographies and bearing area curves (BACs) obtained in the longitudinal turning process for all the analyzed cutting trials. On the surface topography maps presented, a varied surface topography can be seen, which is dependent on the feed value adopted in the research.

**Figure 10:**
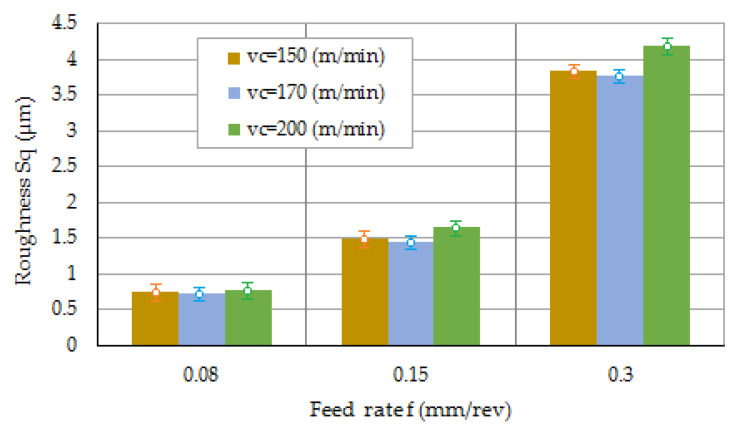
The effect of FR on the Sq parameter.

Figure 10 presents the effect of FR on the roughness parameter Sq. In the current work, the Sa value was obtained in the range of 0.73–4.19 μm. Kadi [27] obtained the optimal surface roughness (Ra = 1.664 μm) during dry cutting of 316L steel. Furthermore, Yasir et al. [43] achieved a value of Ra = 0.537 μm when milling AISI 316L steel. It was found that a lower surface roughness value can be obtained at lower FR values (Figure 10). The effect of the CS on the Sq parameter is marginal.

It has been found that as the CS increases, especially at lower FRs, chips are removed much faster. This provides less time for sufficient heat dissipation.

**Figure 11:**
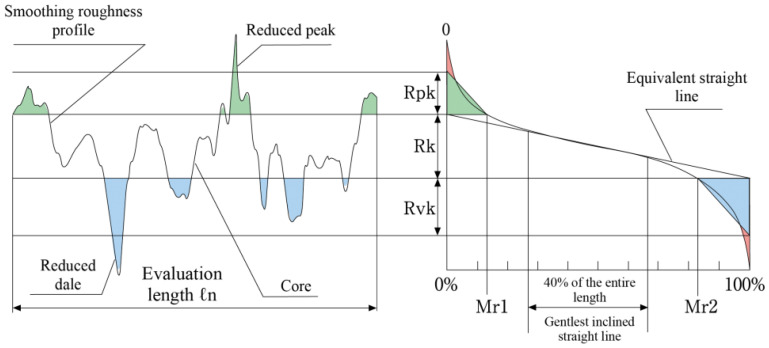
Parameters of a surface with stratified functional properties.

At small FR values (0.08 mm/rev) a built-up layer forms on the machined surface with increasing CS. The thickness of built-up layer decreases with decreasing CS. Based on the BAC, we can describe the functional behavior of the surface using Rvk (reduced valley depth) parameters Rpk and Rk (Figure 11).

Reduced peak height Rpk and the profile depth of the roughness core Rk are parameters defining the profile depth of the roughness core. The Rpk parameter determines the behavior of the geometric structure of the surface during lapping elements [44]. Based on the value of the Rvk parameter, it can be concluded the geometric surface structure has the ability to maintain a lubricant film. Surfaces requiring good lubrication should have high values of reduced valley depth [44]. Based on the BACs obtained, it can be concluded that the most abrasion-resistant surfaces are those machined with low values for both the feed rate and the cutting speed.

### 3.3. Optical Microscopy

**Figure 12:**
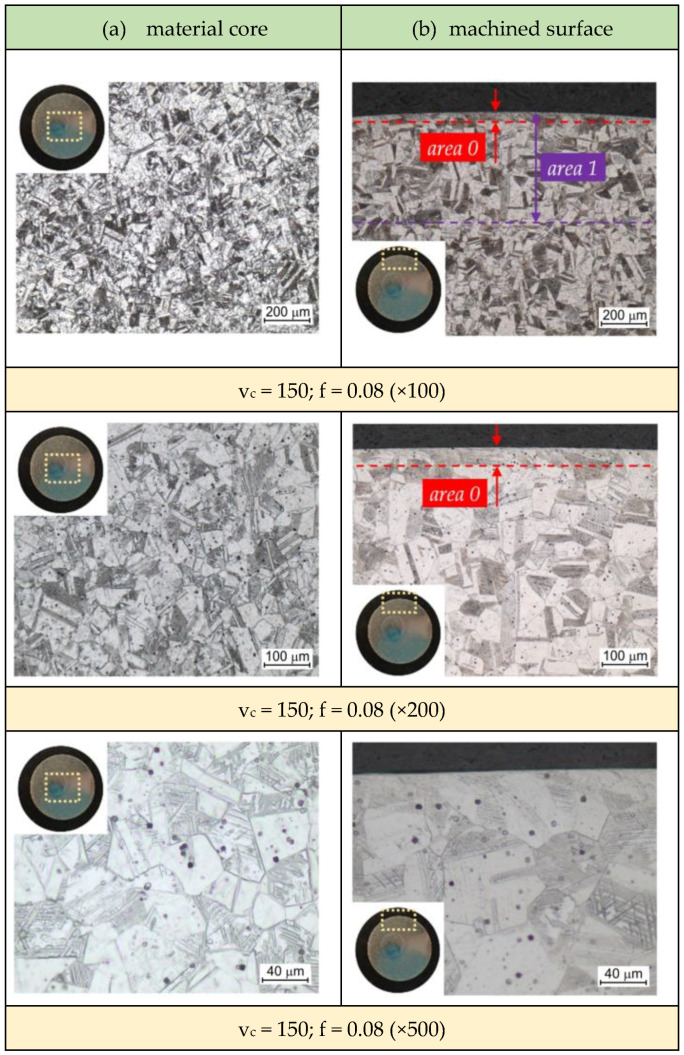

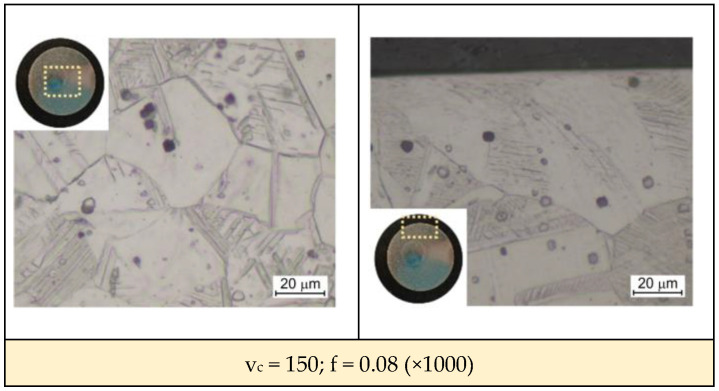
AISI 316L steel microstructure: (**a**) core material; (**b**) machined surface (units: v_c_—m/min, f—mm/rev).

When analyzing the evolution of the microstructure during longitudinal turning, one should take into account changes in the temperature of this layer during the machining process. The tendency of austenite to grain growth depends not only on the temperature but also on the heating time. Grain growth occurs during heating at a temperature higher than the recrystallization temperature of AISI 316L steel (approx. 600 °C). The gradient of changes in the microstructure caused by longitudinal turning was assessed by optical microscopy. Figure 12 presents changes in the microstructure of the material subjected to the longitudinal turning with parameters corresponded for experiment 1 (Table 3). The changes is microstructure of the deformed layer (area 1) are observed to a depth of approximately 400 μm. Near the surface layer, a layer (area 0) with a width of approximately 30 μm was found, in which the grains were strongly deformed. Moreover, the presence of slip bands directed in the direction of feed was observed. In addition, at a depth of 30 to approximately 350 μm, the grains were no refined, but their orientation was very diversified; slip bands are not visible here. With the increase in distance from the surface layer, the change in orientation of slip bands gradually decay. At a depth of approximately 400 μm, the change in orientation within the grains was almost imperceptible. The grains that were closer to the treated surface were subjected to greater deformations.

**Figure 13:**
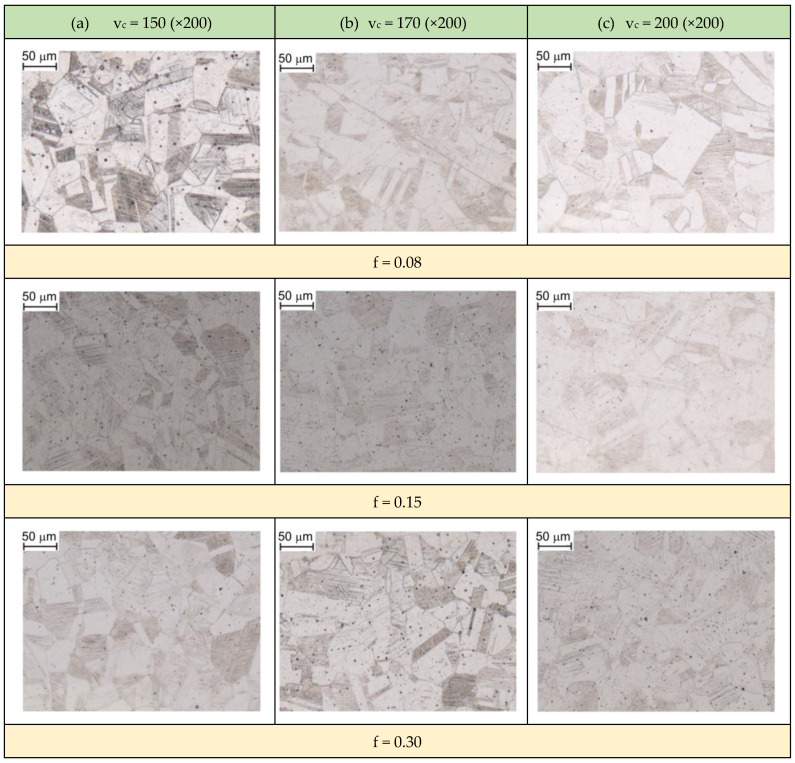
Microstructural evolution of 316L stainless steel (units: v_c_—m/min, f—mm/rev).

The strongly deformed “area 0” (Figure 12) is characterized by a high density of slip bands and twin deformations. Twinning deformation (Figure 13) occurs due to strong plastic strains at low temperature. Twinning mechanism also occurs at a high strain rate and at high temperature. Therefore, the twinning during cutting is a result of very high strain rate at elevated temperature or cold strain near the surface layer.

It was observed that the grain size in the material in as-received state, not subjected to the longitudinal turning, was approximately 27 μm. After the turning process, new grains with larger sizes were formed and the existing grains grew along with the increase in the time of impact of the generated heat in the machined surface. The average grain size g_s_ of samples presented in Table 4 was measured in accordance with ASTM E112 standard.

**Figure 14:**
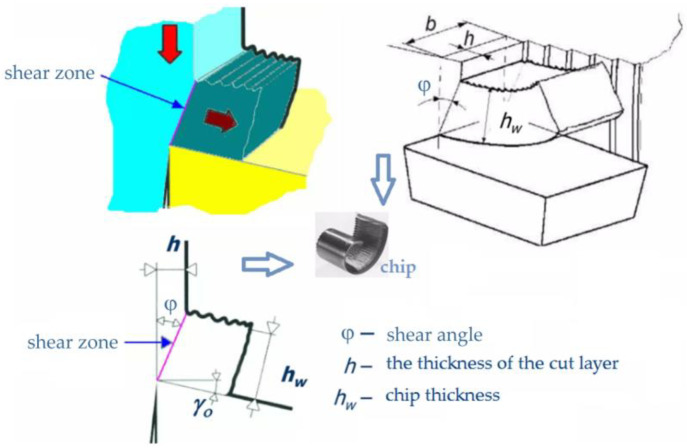
Shear angle of chip thickness ratio.

An important parameter of the cutting process characterizing the amount of deformation in the shear zone is the shear angle φ between the shear zone and direction of the CS. The smaller angle φ is, the greater the length of the shear zone (Figure 14).

It should be emphasized that the shear angle also affects the chip thickness *h*_w_. The ratio of this thickness to the thickness of the machined layer (*h*) is called the cutting ratio Λ_h_ (Equation (2)). It can be used as a measure of deformation in the shear zone.

(2) $$\Lambda_{h}=\frac{h_{w}}{h}$$

The cutting ratio Λ_h_ has a significant impact on the grain size increase. With the increase in its value (in the range of the examined cutting parameters), grain size growth occurs in the “area 1” zone (Figure 12). In turn, the increase in the value of the Λ_h_ is decisively influenced by the CS. As can be seen (Table 4), at a CS of 150 m/min, the size g_s_ was approximately 31 μm. By increasing the CS to 200 m/min, the average grain size increased to approximately 52 μm. However, there is no effect of the FR on significant changes in grain size.

When machining at a CS of 170 m/min and FRs of 0.08 mm/rev and 0.15 mm/rev, an uniform increase in hardness at a depth of approximately 2 mm from the machined surface clearly dominates (Figure 7). Increased hardness is a result of grain refinement close to the machined surface, martensitic transformation induced by plastic deformation or work hardening phenomenon. A change in material properties of workpiece induced by plastic deformation occurs in a workpiece when it is subjected to a stress even lower than the yield stress of that material. The reason for this transformation is the mechanism of elastic-plastic deformations occurring in the cutting zone during the chip formation process.

### 3.4. Statistical Analysis

The multiple regression model available in the STATISTICA program was used The following Equation was applied:

(3) $$Y=\beta_{0}+\beta_{1}X_{1}+\beta_{2}X_{2}$$

where $\beta_{0}$, $\beta_{1}$, and $\beta_{2}$ are linear parameters of the regression function estimated by the least squares method, Y is dependent variable and Xi are independent variables

**Figure 15:**
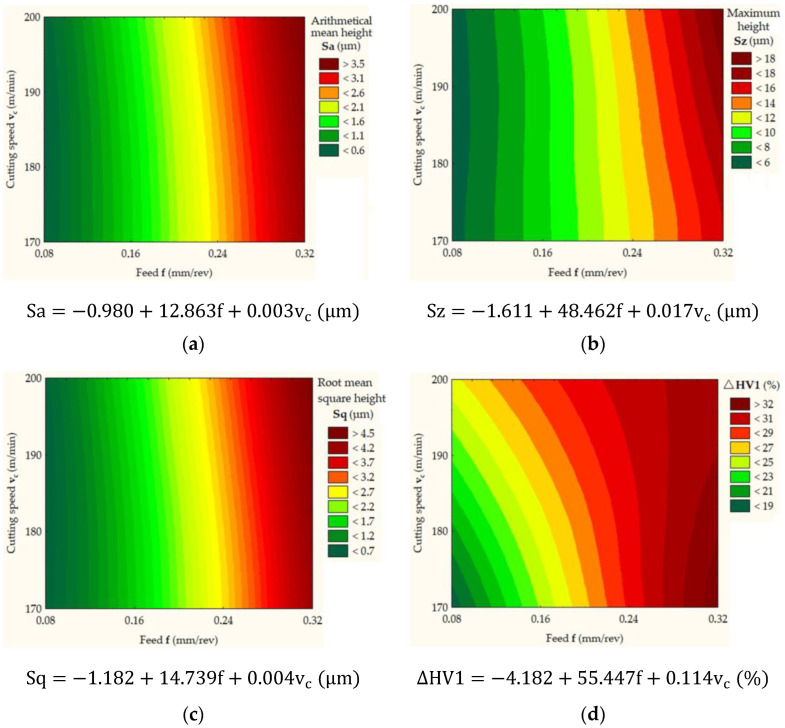
Effect of the FR and CS on the (**a**) Sa parameter, (**b**) the Sz parameter, (**c**) the Sq parameter and (**d**) ΔHV1.

Based on Equation (3), an analysis of the relationship between the values of CS (v_c_) and FR and independent parameters (Sa, Sz, Sq, and ∆HV1) was carried out. As a result of the analysis, multiple regression models (Figure 15) were obtained.

The obtained statistical models describing the Sa, Sz and Sq parameters are characterized by the coefficients of determination R^2^ = 0.991, R^2^ = 0.992 and R^2^ = 0.989, respectively. The standard errors of estimation for ANOVA models are equal to 0.137, 0.705 and 0.172, respectively. The smaller the value of the standard error of the estimation, the better the fit of the model. The values of the coefficients $\beta_{0}$, $\beta_{1}$, and $\beta_{2}$ are shown in Figure 15. The regression coefficients are estimates of the regression coefficients for the entire population. A statistically significant effect of the FR on the Sa, Sz and Sq parameters was observed at the significance level of *p* = 0.000. However, there was no statistically significant effect of the CS on the analyzed dependent variables Sa, Sz and Sq at the significance levels of *p* = 0.254, *p* = 0.185 and *p* = 0.228, respectively. The statistical model describing the change in the parameter ΔHV1 is characterized by the coefficient of determination R^2^ = 0.922 and the standard error of model estimation of 2.591. A statistically significant effect of the CS and FR on the parameter ΔHV1 was observed at the significance levels of *p* = 0.001 and *p* = 0.035, respectively.

### 3.5. Chip Forms

Austenitic stainless steels are susceptible to work hardening phenomenon, and after cold plastic working, their high strength contributes to the rapid wear of cutting tools. However, even in a highly work-hardened state, due to their high ductility, they form long chips that tend to stick to the tool. The occurrence of the built-up edge may increase the cutting force values and, consequently, lead to fracture of the tool. In addition, the thermal conductivity of austenitic steels is approximately three times lower compared to carbon steels, which reduces tool life [45].

**Figure 16:**
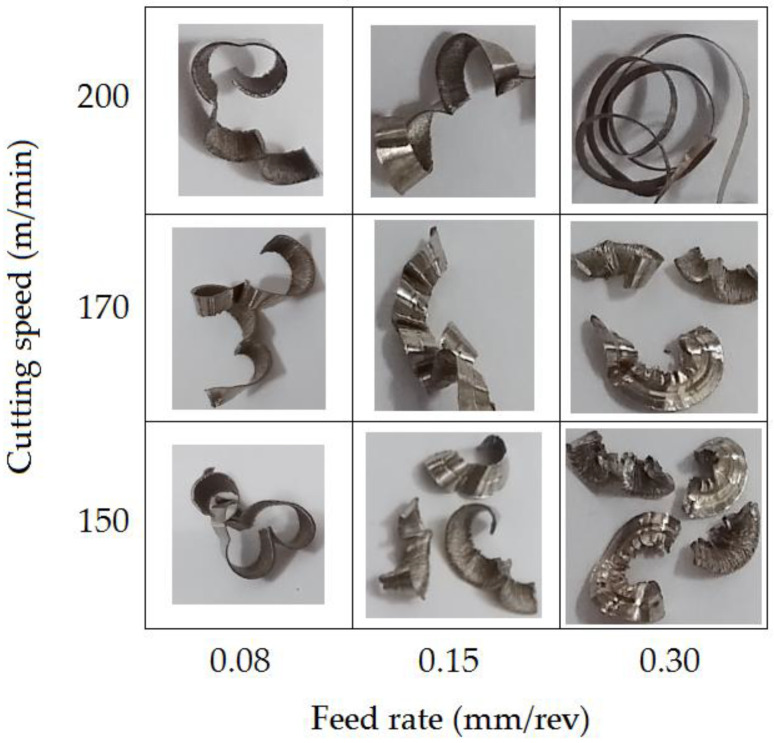
Chip forms.

Changes in the shape of the chips are shown in Figure 16. Three types of chips were observed under dry cutting conditions: tangled, short spiral or long spiral. Chips were classified according to ISO 3685-1993 [46]. In experiment no. 9 (Table 3), a snarled chip (type 1.3 according to [46]) was obtained; in experiments 2, 3 and 6, loose (arc) chips were obtained (type 6.2 according to [46]); in experiments 1, 4, 5 and 7, long connected chips were obtained (type 6.1 according to [46]). A clear boundary between the occurrence of tangled and long spiral chips and short spiral chips was observed at the FR of 0.15 mm/rev. In addition, it was noted that the CS also affects chip formation during AISI 316L longitudinal turning.

As shown in Figure 8a, there is a clear effect of CS on hardness. Hardness increases clearly with increasing speed. For small FR values, and at the same time with increasing CS, the chips take the form of continuous and long chips (Figure 16). Such a chip slows down the process of heat dissipation, because stainless steel has a low value of thermal conductivity. While the thermal conductivity coefficient for structural steel is 50–60 W/(m·K), this coefficient for stainless steel is 13–17 W/(m·K). In turn, higher FRs generate the formation of higher cutting forces in a dominant manner, which in turn translates into greater cutting power, generating increased heat in the cutting zone.

## 4. Conclusions

This work analyses the effect of selected cutting parameters on the integrity of the machined surface of AISI 316L steel. The following conclusions can be drawn from the conducted research:

- Based on the analysis of variance, the feed rate was the most important cutting parameter affecting the parameters Sa, Sz and Sq of the machined surface.
- A statistically significant effect of the CS and the FR on the parameter ΔHV1 was observed.
- The maximum value of hardness is reached at a depth of 20 μm and decreases with the depth of measurement. It was also noted that at a depth of up to 240 μm, the maximum hardness of 270–305 HV1 was reached, hence the height of the machining impact zone can be determined, which is approximately 240 μm for almost all machining conditions.
- The FR affects the cutting ratio significantly, while the influence of the CS is insignificant.
- The cutting ratio Λ_h_ has a significant impact on the grain size increase.
- Decreasing the CS from 200 m/min to 150 m/min resulted in decreasing the average grain size from 52 μm to approximately 31 μm.
- Machined surfaces are characterized by mean roughness in the range of 0.73–4.19 μm.
- The evolution of the microstructure is correlated with the change in the value of the cutting speed.

This work was limited to the assessment and optimization of the impact of selected processing parameters on the surface roughness and hardness of 316L stainless steel. Comparative surface integrity studies are planned for other biocompatible materials. The tests will be performed for different cutting conditions, different tool materials and different cooling conditions.

## Figures and Tables

**Table 1:** Chemical constitution (wt%) of 316L steel (based on spectral analysis).

C	Si	Mn	P	S	Cr	Mo	Ni	Fe
0.03	0.5	1.6	0.035	0.025	17.4	2.2	9.6	balance

**Table 2:** Selected mechanical properties of 316L steel.

Parameter	Value	Unit
Young’s modulus	200,000	MPa
Ultimate tensile strength	485	Mpa
Hardness	210	HV
Heat conductivity	14.6	W/m·K

**Table 3:** Cutting parameters.

Experiment	Cutting Speed v_c_ (m/min)	Feed Rate f (mm/rev)	Depth of Cut a_p_ (mm)
E1	150	0.08	1.5
E2	150	0.15	1.5
E3	150	0.30	1.5
E4	170	0.08	1.5
E5	170	0.15	1.5
E6	170	0.30	1.5
E7	200	0.08	1.5
E8	200	0.15	1.5
E9	200	0.30	1.5

**Table 4:** Average grain size of samples machined with different conditions.

Experiment	v_c_ (m/min)	f (mm/rev)	g_s_ (μm)
1	150	0.08	31.18 ± 1.21
2	150	0.15	31.89 ± 0.45
3	150	0.30	31.13 ± 1.09
4	170	0.08	43.22 ± 2.56
5	170	0.15	44.31 ± 2.54
6	170	0.30	45.89 ± 2.24
7	200	0.08	50.31 ± 1.74
8	200	0.15	51.82 ± 1.67
9	200	0.30	52.55 ± 1.24

## Data Availability

The data presented in this study are available on request from the corresponding author.
